# Asymptomatic contained left ventricular rupture

**DOI:** 10.1186/1532-429X-18-S1-T5

**Published:** 2016-01-27

**Authors:** Ceri Twigg, Jen Bryant, James Shambrook, Stephen Harden, Charles Peebles

**Affiliations:** Cardiothoracic Radiology, University Hospital Southampton U.K., Southampton, United Kingdom

## Background

A 55 year old patient presented late to the emergency department following an ST elevation myocardial infarction (STEMI).

Coronary angiogram demonstrated occluded left anterior descending artery with backfilling from collaterals. The patient was referred for an in-patient cardiac MRI scan (CMRI) to quantify left ventricular (LV) function and viability.

A repeat CMRI was requested 3 months later as an out-patient, to check that LV thrombus demonstrated on the first scan had resolved after treatment.

## Methods

Both investigations used steady-state free precession cine imaging to demonstrate ventricular function. Tissue characterisation and viability assessment was with black blood T1 and T2 weighted images, T1 mapping, early and late post gadolinium enhanced images.

## Results

The in-patient CMRI scan demonstrated aneurysmal dilatation of the distal anterior wall, septum, and apex. Organised thrombus was seen within the apex of the aneurysm. Tissue characterisation sequences suggested extensive oedema over the anterior wall, apex, and basal septum. Early cine images post contrast suggested microvascular obstruction (MVO) over the anterior wall and apex. Late gadolinium enhanced images (LGE) showed full thickness scar through the apex, becoming sub endocardial over the distal anterior wall and septum. As the majority of this scar was greater than 75% thickness, with the associated MVO, residual viability was unlikely.

The 3 month follow-up scan demonstrated a massive LV apical aneurysm. The aneurysm had maximum dimensions of 9.5 cm × 6.6 cm, with a neck measuring 2.4 cm. The aneurysm was larger than the remainder of the LV cavity, with the inferior wall of the LV and septum demonstrating reasonable contractility.

LGE showed enhancement as described in the earlier scan, with new pericardial hyperenhancement.

Due to the size of the aneurysm and morphology, a false aneurysm was suspected. These findings were communicated immediately to the patient's Cardiologist, and the patient was admitted urgently for surgical repair.

## Conclusions

This patient's rapidly developed false aneurysm was diagnosed early enough by CMRI for the patient to undergo surgical repair. CMRI identifies false aneurysms, and quantifies LV function, which allows for post-surgical predictions of LV function.

CMRI is the best technique for imaging LV aneurysms. Function and viability can both be quantified, which is important for surgical planning (Tran, Ross, Colletti and Ching JCMR 2005).

Radiographers and CMR technicians have an important role in appreciating the findings of a false aneurysm, adapting the scan protocol if necessary, as well as highlighting the case to the reporting clinician for their prompt attention.

